# Lectures to General Practitioners on the Diseases of the Stomach and Intestines

**Published:** 1904-11

**Authors:** 


### BOOK REVIEWS.

Lectures to General Practitioners on the Diseases of the Stomach and Intestines. With an account of their relations to Other Diseases, and of the Most Recent Methods Applicable to the Diagnosis and Treatment of them in General; also "The Gastro-intestinal Clinic," in which all Such Diseases are Separately Considered. By Boardman Reed, M.D., Professor of Diseases of the Gastro-intestinal Tract, Hygiene and Climatology in the Department of Medicine of Temple College, Philadelphia. Illustrated. Cloth. Pp. 1021. Price, $5 net. New York: E. B. Treat & Co. 1904.

Coming, as this book does, at a time when the stomach has become the arena of so much vigorous investigation, it will be welcomed by the general practitioner on account of its concise, practical points in the methods of examination, diagnosis and treatment in these diseases.

It has been arranged in the form of eighty-two lectures, thus bringing author and reader more *en rapport* with each other, and includes the anatomy and physiology of the digestive tract, the available methods of examination of the organs, secretions and excretions concerned, and the bearing of the findings upon diagnosis; treatment including diet, massage, exercises, electricity, mechanical vibration, medicinal agents, lavage and other mechanical forms of treatment. This is followed by the gastro-intestinal clinic, which discusses the various diseases separately, and their symptoms, diagnosis and treatment, including one lecture by Dr. Collier Martin on diseases of the rectum and anus.

Dr. Reed was for some years the editor of the *International Medical Magazine*, and through his influence that magazine has contributed largely to the literature bearing on this specialty. He has quoted freely from these articles in the preparation of his book.

The etiology and pathology of the various diseases are treated very briefly, and the book is stript of the discussion of many disputed unsettled questions, thus giving more space for a description of the methods of diagnosis, treatment, etc.

It is rather surprising that so important a subject as Gastric Atrophy or Achylia Gastrica should have been dismissed with only two pages.

However, the volume stands as a ready clinical guide to the elucidation of those perplexing problems which are a part of gastro-intestinal diseases, and the author brings out well those little points which make for or against success in the management of this class of cases.

